# Enhancing Spin-Based Sensor Sensitivity by Avoiding Microwave Field Inhomogeneity of NV Defect Ensemble

**DOI:** 10.3390/nano12223938

**Published:** 2022-11-08

**Authors:** Yulei Chen, Tongtong Li, Guoqiang Chai, Dawei Wang, Bin Lu, Aixin Guo, Jin Tian

**Affiliations:** School of Physics and Information Engineering, Shanxi Normal University, Taiyuan 041004, China

**Keywords:** defect-center materials, Zeeman effect, sensors, quantum optics

## Abstract

The behavior of the magnetic field sensitivity of nitrogen-vacancy (NV) centers as a function of microwave power and the inhomogeneous distribution of MW fields was systematically studied. An optimal structure for exciting spin structures by MW signals was designed using two parallel loop antennas. The volume of the homogeneous regions was approximately 42 mm^3^, and the associated diameter of the diamond reached up to 5.2 mm with 10^16^ NV sensors. Based on this structure, the detection contrast and voltage fluctuation of an optically detected magnetic resonance (ODMR) signal were optimized, and the sensitivity was improved to 5 nT/√Hz. In addition, a pulse sequence was presented to fully eliminate the MW broadening. The magnetic field sensitivity was improved by approximately one order of magnitude as the π-pulse duration was increased to its coherence time. This offers a useful way to improve the sensitivity of spin-based sensors.

## 1. Introduction

The concept of the nitrogen-vacancy (NV) center in a diamond has been employed in many fields including fundamental physics testing [1], solid-state quantum computations [2], solid-state quantum sensing and metrology, and in the field of quartz sensors and graphene oxide wireless magnetic field sensors [3,4]. In particular, the use of NV-based quantum sensors has been demonstrated in a variety of fields such as nuclear magnetic resonance (NMR) technology [5], protein detection [6], magnetometry [7], gyroscopy [8], strain/stress/press sensors [9], electrometry [10], thermometry [11], and others [12,13,14,15].

All of these solid-state quantum sensors take advantage of the principal features of NV centers: all-optical readout and long coherence time at a room temperature of 20 °C [16,17,18]. However, the ground state of the spin structures needs to be excited via microwave (MW), which is the key technology in NV sensors. Especially for the NV center ensemble, not only a single NV center but all NV centers are excited by MW technology to explore higher-precision sensors [19]. Thus, the number of NV centers required to sense a magnetic field depends on the MW technology [20], and the magnetic field sensitivity is limited by the microwave power and inhomogeneous distribution of the MW field.

Here, we focused on using a simplified model to investigate the behavior of the magnetic field sensitivity, detection contrast, and the number of NV sensors as a function of microwave power and the inhomogeneous distribution of the MW field. Based on a theoretical model, the sensitivity of the magnetic field was improved by assembling the diamond in the center of a uniform microwave field. In addition, a pulse sequence was demonstrated to fully eliminate the linewidth broadening caused by the microwave power and inhomogeneous distribution of the MW field. The optimal sensitivity was improved to 0.5 nT/√Hz as the π-pulse duration was increased to its coherence time.

## 2. Model

### 2.1. Magnetic Field Sensitivity

In general, the shot-noise-limited sensitivity of a solid-state NV magnetometer is approximated by [21]

(1) $$S\approx \frac{ћ}{g_{s}\mu_{B}}\frac{1}{R\sqrt{N}}\frac{1}{\sqrt{\eta T_{2}^{*}\tau }}$$

where ħ is the reduced Planck constant; $g_{s}\approx 2$ is the ground state NV-Landé g-factor; μB is the Bohr magneton; *R* is the detection contrast for states with mS = 0 and mS = ±1; and *N* is the number of NV sensors. *η* is the number of photons collected per NV per measurement, which depends on the measurement system. *T*_2_^*^ is the electron spin decoherence time, which is dependent on the quality of the samples, which limits the optimal measurement time *τ*. Hence, the shot-noise-limited sensitivity of magnetic field S can be improved by increasing the number of NV sensors N and the detection contrast R. The number of NV sensors N can be determined by increasing the density of NV defects in the diamond or the size of the NV-holding diamond. In addition, the detection contrast R can be enhanced by increasing the microwave power and homogeneity of the microwave fields.

### 2.2. Model of NV Center Spin Dynamics

The ODMR signals of the NV defect ensemble were modeled and investigated with the parameters of inhomogeneous microwave fields, power broadening, inhomogeneous magnetic fields, and inhomogeneous strain distributions. Microwave signals were applied to excite the NV defects along the z axis. We defined the x axis as an orthogonal direction of the applied microwave. The Hamiltonian of the NV defects is as follows [22,23]:

(2) $$\begin{array}{c} H=ћ\sum_{k=1}^{N}\{D_{k}{\hat{S}}_{z,k}^{2}+g_{s}\mu_{B}B_{d}^{k}{\hat{S}}_{x,k}+E_{1}^{k}\left({\hat{S}}_{x,k}^{2}-{\hat{S}}_{y,k}^{2}\right)+E_{2}^{k}\left({\hat{S}}_{x,k}{\hat{S}}_{y,k}+{\hat{S}}_{y,k}{\hat{S}}_{x,k}\right)+\\ \lambda \cos\left(\omega t\right){\hat{S}}_{x}^{k})+A_{//}\left({\hat{S}}_{x,k}{\hat{i}}_{x,k}\right)+\frac{A_{⊥}}{2}\left({\hat{S}}_{+,k}{\hat{i}}_{-,k}+{\hat{S}}_{-,k}{\hat{i}}_{+,k}\right)+P\left({\hat{I}}_{x,k}^{2}-\frac{1}{3}{\hat{I}}^{2}\right)-g_{n}\mu_{N}B_{z}^{k}{\hat{I}}_{z,k}\} \end{array}$$

where ${\hat{S}}_{k}$(${\hat{I}}_{k}$) denotes a spin operator of electron (nuclear) spin; D denotes zero-field splitting; $g_{s}\mu_{B}B_{d}^{k}{\hat{S}}_{x,k}$ (−$g_{n}\mu_{N}B_{z}^{k}{\hat{I}}_{z,k}$) denotes a Zeeman term of electron (nuclear) spin according to the inhomogeneous magnetic fields; and $E_{1}^{k}$ and $E_{2}^{k}$ denote the inhomogeneous strain distributions along the *x* (*y*) directions. *λ* and *ω* are the microwave amplitude and frequency, respectively, and P denotes the quadrupole splitting. *A*// (*A*⊥) denotes a parallel (orthogonal) hyperfine coupling that detunes the energy of the electron spin.

For small applied magnetic field, because the *x* and *y* components of the magnetic field contribute only to a trivial change in the crystal axis, we considered only the effect of the magnetic field along the *z* axis. Hence, the flip-flop term $\frac{A_{⊥}}{2}\left({\hat{S}}_{+,k}{\hat{i}}_{-,k}+{\hat{S}}_{-,k}{\hat{i}}_{+,k}\right)$ is negligible, and the parallel term $A_{//}\left({\hat{S}}_{x,k}{\hat{i}}_{x,k}\right)$ is dominant. In a rotating frame defined by $U=e^{-i\omega {\hat{S}}_{z}^{2}t/ћ}$, the Hamiltonian is simplified as

(3) $$H≅ћ\sum_{k=1}^{N}\left\{\begin{array}{c} \left(D_{k}-\omega \right){\hat{S}}_{z,k}^{2}+g_{s}\mu_{B}B_{d}^{k}{\hat{S}}_{x}+E_{1}^{k}\left({\hat{S}}_{x,k}^{2}-{\hat{S}}_{y,k}^{2}\right)\\ +E_{2}^{k}\left({\hat{S}}_{x,k}{\hat{S}}_{y,k}+{\hat{S}}_{y,k}{\hat{S}}_{x,k}\right)+\frac{\lambda }{2}{\hat{S}}_{x}^{k}) \end{array}\right\}$$

In general, the number of excited spins was much lower than the total number of spins. The spin ensemble worked as harmonic oscillators such as the bright state ${\hat{b}}_{k}^{*}≅{|B\rangle }_{k}\langle 0|$ and the dark state ${\hat{d}}_{k}^{*}≅{|D\rangle }_{k}\langle 0|$, where ${|B\rangle }_{k}=\frac{1}{\sqrt{2}}\left({|1\rangle }_{k}+{|-1\rangle }_{k}\right)$ and ${|D\rangle }_{k}=\frac{1}{\sqrt{2}}\left({|1\rangle }_{k}-{|-1\rangle }_{k}\right)$. Hence, we can simplify the Hamiltonian as follows:

(4) $$\;H≅ћ\sum_{k=1}^{N}\left\{\begin{array}{c} \left(\omega_{b}^{k}-\omega \right){\hat{b}}_{k}^{*}{\hat{b}}_{k}+\left(\omega_{d}^{k}-\omega \right){\hat{d}}_{k}^{*}{\hat{d}}_{k}+B_{k}\left({\hat{b}}_{k}^{*}{\hat{d}}_{k}+{\hat{d}}_{k}^{*}{\hat{b}}_{k}\right)\\ +iE_{2}^{k}\left({\hat{b}}_{k}^{*}{\hat{d}}_{k}-{\hat{d}}_{k}^{*}{\hat{b}}_{k}\right)+\frac{\lambda }{2}\left({\hat{b}}_{k}+{\hat{b}}_{k}^{*}\right) \end{array}\right\}$$

where $\omega_{b}^{k}=D_{k}-E_{1}^{k}$, $\omega_{d}^{k}=D_{k}+E_{1}^{k}$, and $B_{k}=g_{s}\mu_{B}B_{z}^{k}$. The dynamics of the NV defects are described by the Heisenberg equation.

(5) $$\frac{d{\hat{b}}_{k}}{dt}=-i\left(\omega_{b}^{k}-i\Gamma_{b}\right){\hat{b}}_{k}-iB_{k}{\hat{d}}_{k}+E_{2}^{k}{\hat{d}}_{k}-i\lambda$$

(6) $$\frac{d{\hat{d}}_{k}}{dt}=-i\left(\omega_{d}^{k}-i\Gamma_{d}\right){\hat{d}}_{k}-iB_{k}{\hat{b}}_{k}+E_{2}^{k}{\hat{b}}_{k}$$

where $\Gamma_{b}\;$($\Gamma_{d}$) denotes the width of the homogeneous broadening for an individual NV center. Considering the effect of an inhomogeneous microwave field, the corresponding Heisenberg equations of motion are then given by

(7) $$\langle {\hat{b}}_{k,\;t=\infty }^{*}{\hat{b}}_{k,\;t=\infty }\rangle ={\left|\frac{\lambda_{k}\left(\omega -\omega_{d}^{k}+i\Gamma_{d}\right)}{\left(\omega -\omega_{b}^{k}+i\Gamma_{b}\right)\left(\omega -\omega_{d}^{k}+i\Gamma_{d}\right)-\left({\left|B_{k}\right|}^{2}+{\left|E_{2}^{k}\right|}^{2}\right)}\right|}^{2}$$

(8) $$\langle {\hat{d}}_{k,\;t=\infty }^{*}{\hat{d}}_{k,\;t=\infty }\rangle ={\left|\frac{\lambda_{k}\left(B_{k}-iE_{2}^{k}\right)}{\left(\omega -\omega_{b}^{k}+i\Gamma_{b}\right)\left(\omega -\omega_{d}^{k}+i\Gamma_{d}\right)-\left({\left|B_{k}\right|}^{2}+{\left|E_{2}^{k}\right|}^{2}\right)}\right|}^{2}$$

where the intensity of microwave amplitude λ differs in different regions of the diamond, so the number of excited NV defects is different. We define Pb (Pd) as the average probability of the NV defects in the bright (dark) state.

(9) $$P_{b}=\sum_{k=1}^{N}\frac{{\left|\frac{\lambda_{k}\left(\omega -\omega_{d}^{k}+i\Gamma_{d}\right)}{\left(\omega -\omega_{b}^{k}+i\Gamma_{b}\right)\left(\omega -\omega_{d}^{k}+i\Gamma_{d}\right)-\left({\left|B_{k}\right|}^{2}+{\left|E_{2}^{k}\right|}^{2}\right)}\;\right|}^{2}}{N}$$

(10) $$P_{d}=\sum_{k=1}^{N}\frac{{\left|\frac{\lambda_{k}\left(B_{k}-iE_{2}^{k}\right)}{\left(\omega -\omega_{b}^{k}+i\Gamma_{b}\right)\left(\omega -\omega_{d}^{k}+i\Gamma_{d}\right)-\left({\left|B_{k}\right|}^{2}+{\left|E_{2}^{k}\right|}^{2}\right)}\;\right|}^{2}}{N}$$

Because the inhomogeneity of microwave amplitude *λ* is independent from the inhomogeneity of $\omega_{b}^{k}$, $\omega_{d}^{k}$, $B_{k}$, and $E_{2}^{k}$, the probabilities are solved as follows:

(11) $$\begin{array}{c} P_{b}≅\frac{1}{N}\sum_{j=1}^{m}{\left|\lambda_{j}\right|}^{2}\sum_{k=1}^{floor\left(\frac{N}{m}\right)}{\left|\frac{\lambda_{k}\left(\omega -\omega_{d}^{k}+i\Gamma_{d}\right)}{\left(\omega -\omega_{b}^{k}+i\Gamma_{b}\right)\left(\omega -\omega_{d}^{k}+i\Gamma_{d}\right)-\left({\left|B_{k}\right|}^{2}+{\left|E_{2}^{k}\right|}^{2}\right)}\;\right|}^{2}=\\ \left(\frac{1}{m}\sum_{j=1}^{m}{\left|\lambda_{j}\right|}^{2})(\frac{m}{N}\sum_{k=1}^{floor\left(\frac{N}{m}\right)}{\left|\frac{\lambda_{k}\left(\omega -\omega_{d}^{k}+i\Gamma_{d}\right)}{\left(\omega -\omega_{b}^{k}+i\Gamma_{b}\right)\left(\omega -\omega_{d}^{k}+i\Gamma_{d}\right)-\left({\left|B_{k}\right|}^{2}+{\left|E_{2}^{k}\right|}^{2}\right)}\;\right|}^{2}\right) \end{array}$$

(12) $$\begin{array}{c} P_{d}≅\frac{1}{N}\sum_{j=1}^{m}{\left|\lambda_{j}\right|}^{2}\sum_{k=1}^{floor\left(\frac{N}{m}\right)}{\left|\frac{\lambda_{k}\left(B_{k}-iE_{2}^{k}\right)}{\left(\omega -\omega_{b}^{k}+i\Gamma_{b}\right)\left(\omega -\omega_{d}^{k}+i\Gamma_{d}\right)-\left({\left|B_{k}\right|}^{2}+{\left|E_{2}^{k}\right|}^{2}\right)}\;\right|}^{2}=\\ \left(\frac{1}{m}\sum_{j=1}^{m}{\left|\lambda_{j}\right|}^{2})(\frac{m}{N}\sum_{k=1}^{floor\left(\frac{N}{m}\right)}{\left|\frac{\lambda_{k}\left(B_{k}-iE_{2}^{k}\right)}{\left(\omega -\omega_{b}^{k}+i\Gamma_{b}\right)\left(\omega -\omega_{d}^{k}+i\Gamma_{d}\right)-\left({\left|B_{k}\right|}^{2}+{\left|E_{2}^{k}\right|}^{2}\right)}\;\right|}^{2}\right) \end{array}$$

Therefore, the detection contrast for states with m_S_ = 0 and m_S_ = ±1 is

(13) $$R=\frac{P_{d}}{P_{b}+P_{d}}=\frac{{\left|B_{k}-iE_{2}^{k}\right|}^{2}}{{\left|\omega -\omega_{d}^{k}+i\Gamma_{d}\right|}^{2}+{\left|B_{k}-iE_{2}^{k}\right|}^{2}}=\frac{{\left|g_{s}\mu_{B}B_{z}^{k}-iE_{2}^{k}\right|}^{2}}{{\left|\omega -D_{k}-E_{1}^{k}+i\Gamma_{d}\right|}^{2}+{\left|g_{s}\mu_{B}B_{z}^{k}-iE_{2}^{k}\right|}^{2}}$$

Hence, the detection contrast R is sensitive to the parameters ${\delta \Gamma }_{d}\;$(inhomogeneous microwave fields and power broadening), $\delta B_{k}$ (inhomogeneous magnetic fields), and $\delta E_{1}^{k}$ and $\delta E_{2}^{k}$ (inhomogeneous strain distributions).

However, for a magnetic sensor based on the NV center ensemble in a diamond, when the diamond sample has been determined to be the sensing element, the feature of inhomogeneous strain distribution is frozen-in, so the associated parameters of $\delta E_{1}^{k}$ and $\delta E_{2}^{k}$ are negligible. Hence, ${\delta \Gamma }_{d}$ is dominant and affects the detection contrast R for the sensitivity of the magnetic sensor. That is, the sensitivity of the magnetic sensor depends significantly on the microwave field and power broadening.

### 2.3. Simplified Model of NV Center Spin Dynamics

We developed a toy model to infer the behavior of the detection contrast R as a function of the microwave field and power broadening. For this purpose, we considered the NV center as a simple closed two-level system, denoted as |0〉 and |−1〉 and corresponding to the spin projections m_s_ = 0 and m_s_ = −1, respectively. The Hamiltonian is given by [23]:

(14) $$H=ћ\omega_{0}|1\rangle \langle 1|+ћ{Ω}_{R}\cos\left(\omega_{m}t\right)\left(|0\rangle \langle 1|+|1\rangle \langle 0|\right)$$

where $\omega_{0}$ is the Bohr frequency and $\;{Ω}_{R}$ is the Rabi frequency. Within this simplified framework, we did not consider the populations in the excited state and metastable state. The spin polarization in the metastable state is described with a polarization rate Γ*_p_*, which follows a standard saturation behavior having optical pumping power P_0_. P_sat_ is the saturation power, and the polarization rate Γ*_p_* can be given by

(15) $$\Gamma_{P}=\Gamma_{P}^{\infty }\times \frac{z}{1+z}$$

where *z* = P_0_/P_sat_ is the saturation parameter of the radiative transition, and $\Gamma_{P}^{\infty }$ is the polarization rate at saturation. Due to the electron spin coherences, the relaxation rate of the coherences is given by

(16) $$\Gamma_{r}=\Gamma_{r}^{\infty }\times \frac{z}{1+z}$$

where $\Gamma_{r}^{\infty }$ is the rate of the optical/microwave cycles at saturation. Using the above notations, the detection contrast R can then be evaluated as

(17) $$R=\frac{I\left(0,\;0,\;s\right)-I\left({Ω}_{R},\omega_{0},\;s\right)}{I\left(0,\;0,\;s\right)}$$

where I(0, 0, s) [resp. $I\left({Ω}_{R},\omega_{0},\;s\right)$] denotes the PL rate of the NV defects without applying the microwave field. Hence, the detection contrast R can be given by

(18) $$R=m\times \frac{{Ω}_{R}{}^{2}}{{Ω}_{R}{}^{2}+\Gamma_{P}^{\infty }\Gamma_{r}^{\infty }{\left(\frac{z}{1+z}\right)}^{2}}$$

where *m* = (*α* − *β*)/2*α* appears as an overall normalization factor. The detection contrast R evolves in the opposite way with the Rabi frequency ${Ω}_{R}=A\sqrt{P_{mw}\times \sigma /\Delta E}\propto \sqrt{P_{mw}}$. The detection contrast R decreases as the average microwave power $P_{av}$ decreases:

(19) $$R\propto \frac{P_{av}}{P_{av}+\Gamma_{P}^{\infty }\Gamma_{r}^{\infty }{\left(\frac{z}{1+z}\right)}^{2}}=\frac{1}{1+\frac{\Gamma_{P}^{\infty }\Gamma_{r}^{\infty }{\left(\frac{P_{0}}{P_{0}+P_{\mathrm{sat}}}\right)}^{2}}{P_{mw}}}=\frac{1}{1+\frac{a}{P_{mw}}}$$

where $a=\Gamma_{P}^{\infty }\Gamma_{r}^{\infty }{\left(\frac{P_{0}}{P_{0}+P_{\mathrm{sat}}}\right)}^{2}$. $\Gamma_{P}^{\infty }$ is fixed at 5 × 10^6^ s^−1^, by the lifetime of the metastable state, which is of the order of 200 ns at room temperature. In addition, $\Gamma_{r}^{\infty }$ is approximately 8 × 10^7^ s^−1^, as set by the excited-state radiative lifetime. In actual applications, when the diamond sample is determined, the parameters $\Gamma_{P}^{\infty }$, $\Gamma_{r}^{\infty }$, $P_{0}$, and $P_{\mathrm{sat}}$ are fixed values, and the associated a is constant. Hence, the detection contrast R can be simplified as

(20) $$R\propto P_{mw}$$

The number of NVs in the NV center is also dependent on the microwave power $P_{mw}$. Hence, based on Equation (20), the shot-noise-limited sensitivity of a solid-state NV magnetometer is approximately given by

(21) $$S\approx \frac{ћ}{g_{s}\mu_{B}}\frac{1}{R\sqrt{N}}\frac{1}{\sqrt{\eta T_{2}^{*}\tau }}\propto \frac{ћ}{g_{s}\mu_{B}}\times \frac{1}{P_{mw}}\times \frac{1}{\sqrt{P_{mw}}}\times \frac{1}{\sqrt{\eta T_{2}^{*}\tau }}$$

In general, the parameters g_s_ and μ_B_ are constants. *η* depends on the measurement system. *T*_2_* depends on the quality of the samples, which limits the optimal measurement time *τ*. Hence, based on Equation (21), the shot-noise-limited sensitivity of a solid-state NV magnetometer can be simplified as

(22) $$S\propto M\frac{1}{P_{mw}}\times \frac{1}{\sqrt{P_{mw}}}=M{\left(\frac{1}{P_{mw}}\right)}^{3/2}$$

where *M* is a constant. Equation (22) shows that the shot-noise-limited sensitivity mainly depends on the microwave power $P_{mw}$. In general, the NV center is excited by the MW antenna. However, due to the non-uniform distribution of the MW field around the MW antenna, the actual power exciting the NV center is not the input power $\Delta P_{in}$. The actual power $P_{mw}$ can be given as

(23) $$P_{mw}=P_{in}-P_{in}\times \sigma$$

where *σ* is the inhomogeneous distribution of the MW field. Hence, the shot-noise-limited sensitivity of a solid-state NV magnetometer can be given as

(24) $$S\propto M{\left(\frac{1}{P_{in}\left(1-\sigma \right)}\right)}^{3/2}$$

## 3. Experimental Setup

The single-crystal bulk diamond (Element Six, Ascot, UK) has an NV density of 10^18^ cm^−3^ with dimensions of 5 mm × 5 mm × 0.5 mm. The NV-center ensemble was fabricated by electron irradiation at 10 MeV for 4 h and annealed at 850 °C for 2 h [24].

The optically detected magnetic resonance (ODMR) signals of the nitrogen vacancy center in the diamond were examined by a confocal microscope system in a constant-temperature environment [25]. A magnetic field was applied along the (111) crystal axis. In this case, the Zeeman splitting of the NV center’s electron structure along the (111) crystal axis was larger than that along the other three crystal axes.

**Figure 1:**
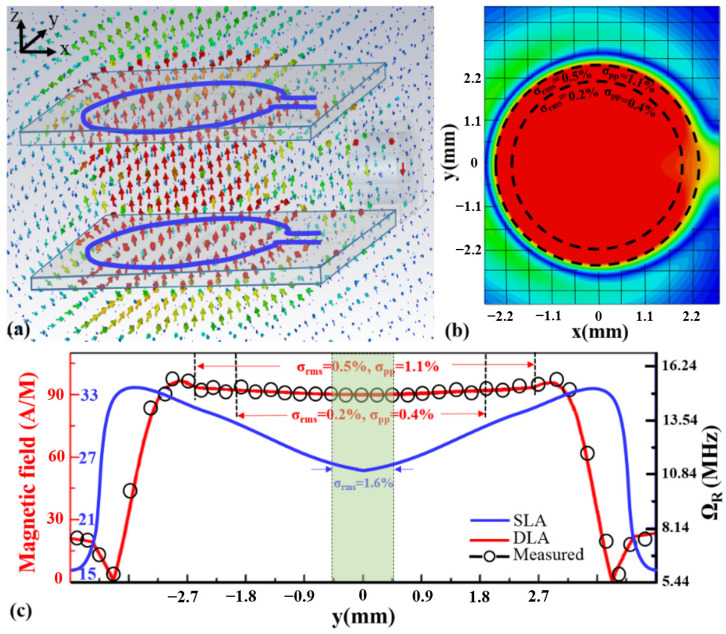
Construction and measurement of the homogeneous microwave field. (**a**) Homogeneous microwave field produced between the two parallel Ω–shaped microwave antenna structures. (**b**) Simulation of the field distribution. (**c**) The magnetic field in a different area.

A homogeneous microwave field was constructed using two parallel Ω-shaped antennas which are in series, as shown in Figure 1a. Compared to the non-uniform distribution microwave field distribution of the single straight copper wire, which is distributed along the axis, the microwave field distribution of the Ω loop antenna utilized here can be concentrated on the center. The diameter of the loop antenna was 8.3 mm, and the associated length of the antenna was 26.13 mm, which was a quarter of the microwave excitation wavelength of 2.87 GHz. The width and thickness of the copper wire were both 40 μm. The distance between the two loop antennas was 2 mm, which could be changed to control the uniformity of the MW field along the z axis. We now experimentally determine how the sensitivity of a solid-state NV magnetometer evolves with the inhomogeneous microwave power *P_av_*.

Based on a theoretical model, a homogeneous microwave field was designed with two microwave antenna structures in the shape of Ω, as shown in Figure 1a. The uniformity of the microwave field distributed in the center of the two antenna structures can be changed with the distance between the double-loop antennas (DLAs). The homogeneity performance was modeled and simulated using a commercial finite element MW simulation software (HFSS). In Figure 1b, the maximum intensity of the MW field is indicated in red, and the minimum is indicated in blue. Within the center of the microwave antennas, the intensity of the MW field was at its maximum, and the distribution of the MW field was uniform. However, at the edge of the center of the microwave antennas, the intensity of the MW field decreased, and the distribution was not uniform.

The uniformity of the microwave field was quantitatively characterized using both the fractional root-mean-square inhomogeneity σ_rms_ and the fractional peak-to-peak variation σ_pp_ = (B_max_ − B_min_)/B_ave_. The two parameters can be used to evaluate and design excellent structures. As shown in Figure 1b, simulations within a 21-mm^2^ circular area centered in the two microwave antennas indicated σ_rms_ = 0.5% and σ_pp_ = 1.1%, whereas simulations in a smaller 12-mm^2^ circular area indicated σ_rms_ = 0.2% and σ_pp_ = 0.4%.

The intensity and homogeneity within the two microwave antennas were evaluated by a standard magnetometer. As shown in Figure 1c, within a 21-mm^2^ circular area centered in the LGR central loop, the measurement results indicated σ_rms_ = 0.5% and σ_pp_ = 1.1%. Within a smaller 12-mm^2^ circular area, measurement results indicated σ_rms_ = 0.2% and σ_pp_ = 0.4%. However, for a single-loop antenna (SLA), within a smaller 0.785-mm^2^ circular area, the nonuniformity was approximately 1.6%, which was worse than that of the DLA. These measurement results are in good agreement with the simulation results in Figure 1b.

### 3.1. Experimental Results

The results showed that the homogeneous MW field was approximately 21 mm^2^, the associated diameter of the diamond was approximately 5.2 mm, and the thickness was approximately 2 mm. In this case, the inhomogeneity of the MW field was very small at approximately 0.5%. As the density of the NV defects was approximately 10^18^ cm^−3^, the number of NV centers was 4.2 × 10^16^, which means that the signal-to-noise ratio could reach up to 10^16^ in theory. In addition, as the homogeneous MW field was approximately 12 mm^2^, the associated diameter of the diamond was approximately 3.9 mm, and the thickness was smaller than 2 mm. Based on the above method, the signal-to-noise ratio can reach up to 10^16^ in theory with the inhomogeneity of the MW field at 0.2%. Meanwhile, the inhomogeneity of the microwave power can be controlled by increasing the distance between the two parallel microwave antenna structures. This can be used to investigate the evolution rule of the sensitivity of a solid-state NV magnetometer with inhomogeneous microwave power.

To further explore the behavior of the sensitivity, detection contrast, the number of NV sensors as a function of the microwave power and the inhomogeneity of the microwave power, a systematic measurement was conducted. A diamond with dimensions of 5 mm × 5 mm × 0.5 mm was assembled in the center of the DLA, and the diameter of the laser beam was set at approximately 1 mm. A similar experiment was implemented using the SLA to compare and analyze the influence of the inhomogeneity and intensity of the MW field.

**Figure 2:**
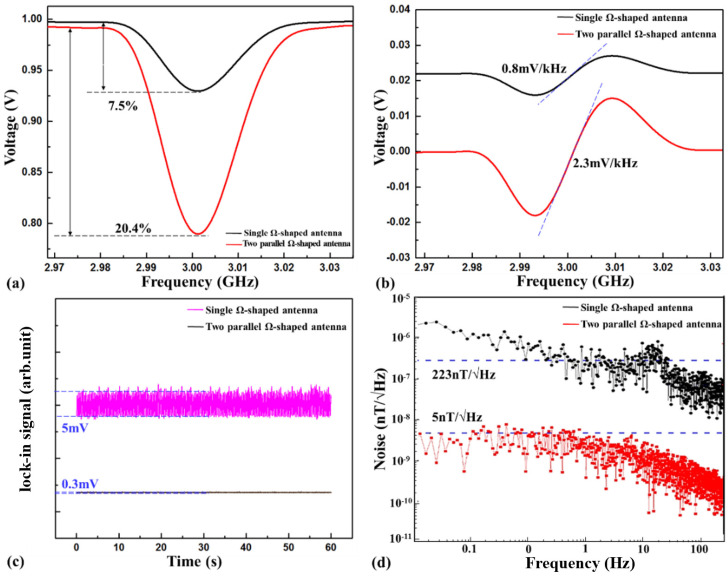
The ODMR signals and the magnetic field sensitivity using the single Ω-shaped antenna and the two parallel Ω-shaped antenna. (**a**) The detection constant R. (**b**) The slope (refer to the sensitivity of spin-based sensors). (**c**) The lock-in signals. (**d**) The bias stability.

As anticipated, the detection constant R was approximately 7.5% using the SLA and approximately 20.4% using the DLA, as depicted in Figure 2a. The detection constant R improved by approximately 2.7 times. The reason can be found in Figure 2c. The intensity of the MW at the center of the DLA was approximately 3.5 times stronger than that at the center of the SLA, as shown in Figure 1c. The measurement result was in reasonable agreement with the model, as illustrated by Equations (18)–(20). The associated coefficient of sensitivity was calculated by the slope using the differential scheme. The slope improved to 2.3 mV/kHz using the DLA, as shown in Figure 2b, which was in reasonable agreement with the model as illustrated by Equation (21). The shot-noise-limit sensitivity increased as the microwave power improved.

The simplified model of the NV center spin dynamics does not allow for the extraction of precise values of the photophysical parameters (e.g., $\Gamma_{P}^{\infty }$ and $\Gamma_{r}^{\infty }$). The two-level toy model was thus sufficient to explain the behavior of the detection constant and sensitivity as a function of the inhomogeneous distribution of the MW field. As shown in Figure 2c, the ODMR signals were locked-in using modulation and demodulation technology. The locked-in position of the spin magnetic resonance curve was the lowest point of the curve. Hence, the voltage fluctuation of the lock-in signal was associated with the ODMR signals, which fluctuated with the inhomogeneity distribution of the MW field.

As depicted in Figure 2c, the voltage fluctuation of the lock-in signal was approximately 5 mV when using the SLA, but only approximately 0.3 mV when using the DLA. Therefore, the stability of the ODMR signals excited by the DLA improved by approximately 16 times. The reason mainly depended on the inhomogeneity distribution of the MW field of two different antennas, as simulated in Figure 1c. The non-uniformity of the MW field at the center of the DLA was far less than that at the center of the SLA.

In order to better explain the behavior of the magnetic field sensitivity of nitrogen-vacancy (NV) centers S as a function of the microwave power *P_in_* (typically the MW magnetic field amplitude for antenna [25]) and the inhomogeneous distribution of the MW fields σ (as shown in Equation (24)), based on the measurements above, we conducted sensitivity tests on SLA and DLA, respectively. For our experimental system, the inhomogeneous distribution of the MW fields σ were 0.2% and 1.6%, respectively, as above-mentioned. Then, the MW magnetic field amplitude was detected on the antenna surface and were 5.3A/m for SLA and 64.1A/m for DLA, respectively. This means that the parameter ${\left[P_{in}\left(1-\sigma \right)\right]}^{-3/2}$ for DLA was 42.9 times than that of SLA. Meanwhile, the sensitivity based on SLA was 223 nT/√Hz and on DLA, it was 5 nT/√Hz, as shown in Figure 2d. This means that the sensitivity based on DLA was approximately 44.6 times that obtained based on the SLA. This measurement result is in reasonable agreement with the model as illustrated by Equation (24). The slightly error mainly comes from the microwave power fluctuation caused by the system thermal noise. It can be seen from the result that the sensitivity was improved by increasing the uniformity of the MW field and MW power. The reason was that (i) the detection contrast quickly decreased based on the inhomogeneous microwave power, and (ii) the linewidth broadened with the inhomogeneous microwave power.

### 3.2. Magnetic Field Sensitivity Using Pulse Sequence

Based on the above results, we demonstrated a method to fully eliminate the linewidth broadening caused by the microwave power and inhomogeneous distribution of the MW field. The time-resolved PL during a read-out laser pulse for the NV center assembly was investigated. If the initial state was prepared in state |1〉 by applying the microwave π-pulse, then the time-resolved PL signal rapidly decayed to the metastable state and then decayed to the |0〉 state. Correspondingly, the PL decayed to the steady-state value within the lifetime of the metastable state. Hence, the microwave power broadening can be fully eliminated by using pulses in a dark condition. 

**Figure 3:**
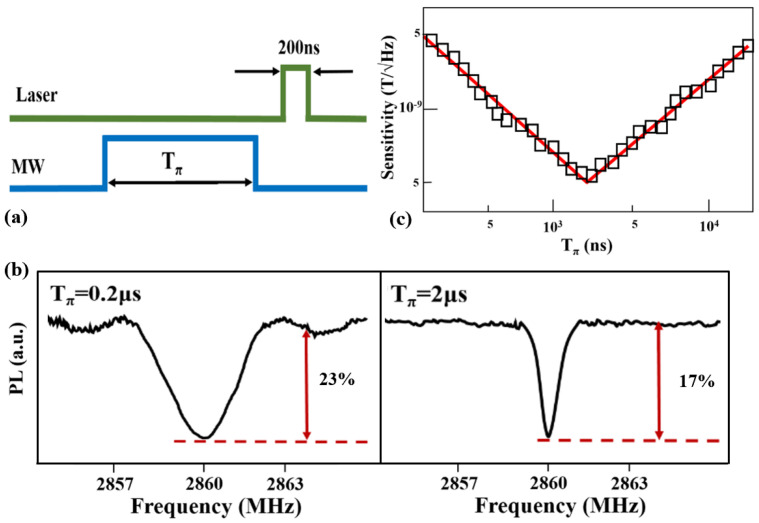
The magnetic field sensitivity using pulse sequence. (**a**) Pulse sequence used to suppress power broadening effects. (**b**) Magnetic resonance spectrum recorded at the excited-state for different duration *T*_π_. (**c**) An enhancement of magnetic field sensitivity by one order-of-magnitude.

The duration of the laser pulse was set to *T_laser_* = 200 ns. A microwave π-pulse followed the laser pulse by 1 µs to ensure the relaxation of steady-state populations trapped in the metastable state. As the π-pulse was applied, the spin rotated in a dark condition, and the power broadening was fully cancelled. Then, the ODMR signals were recorded by continuously repeating the sequence. As shown in Figure 3a, a pulse sequence was designed to eliminate the MW power broadening. In order to obtain the optimal π-pulse duration, the linewidths of the ODMR signals were investigated. As depicted in Figure 3b, the horizontal axis was the MW frequency. The linewidth was approximately 2.5 MHz when *T_π_* = 0.2 μs. As the π-pulse duration increased, the linewidth became sharper, but the detection contrast decreased. In this case, the results indicated that the linewidth broadening (caused by the microwave power and inhomogeneous distribution of the MW field) was fully cancelled in the experiment. 

Hence, to explore the optimal shot-noise limited sensitivity of a magnetic field, the sensitivity was measured by increasing the π-pulse duration according to Equation (1). As depicted in Figure 3c, as the π-pulse duration increased, the sensitivity increased. As the duration time Tπ of the π-pulse increased to its coherence time *T*_2_* = 2.0 ± 0.1 μs, the sensitivity improved to an optimal value of 0.5 nT/√Hz. In this situation, the MW broadening was fully cancelled. As we continued to increase the π-pulse duration, the sensitivity began to decrease. This was because the detection contrast significantly decreased. The results show that the microwave power broadening was cancelled by the pulse sequence, and the magnetic field sensitivity was improved by one order of magnitude based on the DLAs. Furthermore, the sensitivity could be further improved by using a Ramsey-type sequence.

## 4. Conclusions

Sensors based on diamond NV color center have demonstrated the potential of ~fT/√Hz ultra-high sensitivity [21,23,24]. Researchers have tried to improve sensitivity through different methods [19,25]. Compared with the single color center sensing method [26], we demonstrated a theoretical model to create a relationship between the sensitivity S, detection constant R, number of NV sensors N, and the uniform distribution of an MW field. This further clarifies the influencing factor of the sensitivity of NV center sensors, and offers a theoretical guide to design optimal structures to excite spin structures by MW signals.

Unlike the traditional microwave radiation mode of the wire antenna [19,21], we designed a homogeneous MW field region with a large volume of 42 mm^3^. A large diamond block was used to sense the magnetic field. The associated diameter of the diamond was approximately 5.2 mm, and the thickness was smaller than 2 mm. The inhomogeneity of the MW field was approximately 0.5%. As the density of the NV defects was approximately 10^18^ cm^−3^, the number of NV centers was 10^16^, which meant that theoretically, the signal-to-noise ratio could reach up to 10^16^. In general, the number of NV centers reported in the review literature was approximately 10^10^ [27]. Hence, based on our method, theoretically the shot-noise-limited sensitivity of a solid-state NV magnetometer can be further improved by an order of magnitude of approximately 10^6^.

For CW-ODMR, the combination of poor readout fidelity combined with an inability to benefit from extended *T*_2_* limited the increase in sensitivity [28]. In contrast to CW-ODMR, pulsed ODMR technique avoids the microwave power and inhomogeneous distribution of the MW field broadening of the spin resonances, enabling nearly *T*_2_* limited measurements. In this study, the proposed methods above were combined to achieve an optimal value of 0.5 nT/√Hz for the NV sensor, which was two orders of magnitude better than the traditional lock-in-only approach [29] and one order of magnitude better than the pulse-only approach [30].

In conclusion, the behavior of the magnetic field sensitivity of NV centers as a function of the microwave power and inhomogeneous distribution of the MW field was studied. The detection contrast was improved by assembling the NV centers into two parallel microwave antenna structures. The voltage fluctuation of the ODMR signal was approximately 0.3 mV, and the sensitivity was improved to 5 nT/√Hz. Then, a pulse sequence was presented to eliminate the MW broadening. As the π-pulse duration was increased to its coherence time, the magnetic field sensitivity improved by approximately one order of magnitude. It was a useful tool to study the weak hyperfine interactions between the NV center and the nuclear spins (^14^N, ^15^N, and ^13^C) in a diamond matrix.

## Data Availability

The data are available on reasonable request from the corresponding author.
